# Supplementary material for: Impact of negative tuberculin skin test on growth among disadvantaged Bangladeshi children
Source: PLoS One. 2019 Nov 7;14(11):e0224752. doi: 10.1371/journal.pone.0224752 (PMC6837307; doi:10.1371/journal.pone.0224752)
Supplement: Attachment — Submitted filename: Response to Reviewers.docx [file pone.0224752.s002.docx]

**Reviewers’ comments to PLOS ONE questions and to the author with author responses:**

Question 1: Is the manuscript technically sound, and do the data support the conclusions?

Reviewer #1: Yes

Response: Thank you.

Reviewer #2: No

Response: Thank you. We have now revised the conclusion in the text for better clarity and hope revised conclusion will qualify for the publication.

Question 2: Has the statistical analysis been performed appropriately and rigorously?

Reviewer #1: Yes

Response: Thank you.

Reviewer #2: Yes

Response: Thank you.

Question 3: Have the authors made all data underlying the findings in their manuscript fully available?

Reviewer #1: Yes

Response: Thank you.

Reviewer #2: No

Response: Thank you. The full dataset was uploaded during the manuscript submission process.

Question 4: Is the manuscript presented in an intelligible fashion and written in standard English?

Reviewer #1: Yes

Response: Thank you.

Reviewer #2: Yes

Response: Thank you.

5. Review Comments to the Author

Reviewer #1: This study has aimed to explore the determinants of TST positivity in children. TB screening as one of the secondary causes of malnutrition was performed on 243 stunted or at-risk of stunting children. Screened children underwent a chest radiograph (CXR), TST, and physical examination for TB diagnosis.

The current study has examined the growth differences between TST-positive and TST-negative undernourished children aged 12 to 18 months who received nutritional intervention prospectively for 90 feeding days.

This is the first study which intended to estimate an impact of TST positivity on children’s growth in response to a community-based nutritional intervention.

Although the current study was designed towards understanding the tuberculosis in malnutrition children, unexpectedly, nothing significant found in the study.

This study should be published in a medical journal for reporting the relationship between tuberculosis and malnutrition in children, but the study is not fit for an international peer-reviewed journal like PLOS ONE.

Response: Thanks for your overall positive comments. Our primary objective was to examine the growth differences between TST-positive and TST-negative undernourished children aged 12 to 18 months who received nutritional intervention prospectively for 90 feeding days in the parent Bangladesh Environmental Enteric Dysfunction (BEED) study. We observed statistically significant improvement of length for age Z-score (LAZ) and weight for age Z-score (WAZ) among the TST-negative participants at the end of intervention period. Our secondary objective was to explore the determinants of TST positivity at enrollment. We observed several factors (maternal age, education, and occupation) that were significantly associated with TST positivity in our study children during univariate logistic regression analysis. However, as you mentioned, multivariable logistic regression failed to identify any association of those factors with TST positivity. As you rightly noticed this is the first study which is intended to estimate an impact of TST positivity on children’s growth in response to a community-based nutritional intervention, and importantly, the objectives of the study are aligned with the methods, results, and conclusion, we feel the revised manuscript bears merit for publication in the PLOS ONE.

Reviewer #2: The manuscript presented no new significant findings. Moreover, it is already known fact to use INH prophylaxis in paediatric populations. The control group lacks significant number of cases to justify and establish the hypothesis raised. The study also documents several limitations, raising questions on study design and case controlling. Outcome is not justified significantly. It is encouraged to increase the study participants and include more objectives rather than only nutritional aspects in paediatric populations.

Response: Thank you. Improved growth velocity in absence of TST positivity and/or active PTB disease below the age of two years is a novel finding which may imply immense public health implications. Moreover, we were unable to ascertain household contact of most of the TST-positive and clinical PTB cases in our study. This observation indicates that a considerable number of undiagnosed adult TB cases may be still available in the community. Contact tracing system is not well conducted in developing countries with a high burden of tuberculosis cases. Thus, our finding underscores the importance to screen the undernourished children for latent pulmonary tuberculosis with or without smear-positive adult household contact who may be benefited from INH prophylaxis, already recommeneded by the WHO. We have revised our conclusion on the basis of your recommendation.

Now, we also calculated the sample size using the formula of a descriptive study: n = Z² p(1-p)/d² and found 163 participants is sufficient for this type of screening program [where, n = required sample size, Z = standard normal variant value for 95% confidence level (1.96), p = expected proportion of the condition (12.4% according to a tuberculin survey conducted in Bangladesh), and d = precision (0.05)]. However, we screened 243 children and we believe the available sample will allow us to perform a case-control analysis between TST-positive and TST-negative participants, nested in the main BEED study. We have incorporated this sample size calculation in the text on page 4.
